# Rationale and design of the multicentric, double-blind, double-placebo, randomized trial APrepitant versus HYdroxyzine in association with cytoreductive treatments for patients with myeloproliferative neoplasia suffering from Persistent Aquagenic Pruritus. Trial acronym: APHYPAP

**DOI:** 10.1186/s13063-021-05864-8

**Published:** 2021-12-19

**Authors:** C. Le Gall-Ianotto, R. Verdet, E. Nowak, L. Le Roux, A. Gasse, A. Fiedler, D. Carlhant-Kowalski, P. Marcorelles, L. Misery, J. C. Ianotto

**Affiliations:** 1 Department of Dermatology, University Hospital of Brest, Brest, France; 2 Univ Brest, LIEN, Brest, France; 3 Research and Innovation Department, University Hospital of Brest, Brest, France; 4 Clinical investigation center, CIC, Inserm 1412, University Hospital of Brest, 29200 Brest, France; 5 Department of Pharmacy, University Hospital of Brest, Brest, France; 6 PPRIGO (Production Pharmaceutique pour la Recherche Institutionnelle du Grand Ouest), Brest coordinator, Brest, France; 7 Pharmacovigilance Unit of Research and Innovation Department, University Hospital of Brest, Brest, France; 8 Brest Biological Resources Center BB-0033-00037, University Hospital of Brest, Brest, France; 9 Department of Pathology, University Hospital of Brest, Brest, France; 10 Department of Clinical Hematology, University Hospital of Brest, Brest, France; 11 France Intergroup of Myeloproliferative neoplasms (FIM), Brest, France

**Keywords:** Aquagenic pruritus, Myeloproliferative neoplasms, Randomized controlled trial, Aprepitant, Hydroxyzine

## Abstract

**Background:**

Aquagenic pruritus (AP), an intense sensation of scratching induced after water contact, is the most troublesome aspect of BCR-ABL1-negative myeloproliferative neoplasms (MPNs). Mostly described in polycythemia vera (PV, ~ 40%), it is also present in essential thrombocythemia (ET) and primary myelofibrosis (PMF) (10%). Even if this symptom can decrease or disappear under cytoreductive treatments, 30% of treated MPN patients still persist with a real impact on the quality of life (QoL). Because its pathophysiology is poorly understood, efficient symptomatic treatments of AP are missing. The neuropeptide substance P (SP) plays a crucial role in the induction of pruritus. Several studies showed the efficacy of aprepitant, an antagonist of SP receptor (NK-1R), in the treatment of chronic pruritus but never evaluated in AP. The objectives of APHYPAP are twofold: a clinical aim with the evaluation of the efficacy of two drugs in the treatment of a persistent AP for MPN patients and a biological aim to find clues to elucidate AP pathophysiology.

**Methods/design:**

A multicentric, double-blind, double-placebo, randomized study will include 80 patients with MPN (PV or ET or PMF) treated since at least 6 months for their hemopathy but suffering from a persistent AP (VAS intensity ≥6/10). Patients will be randomized between aprepitant (80 mg daily) + placebo to match to hydroxyzine OR hydroxyzine (25 mg daily) + placebo to match to aprepitant for 14 days. At D0, baseline information will be collected and drugs dispense. Outcome measures will be assessed at D15, D30, D45, and D60. The primary study endpoint will be the reduction of pruritus intensity below (or equal) at 3/10 on VAS at D15. Secondary outcome measures will include the number of patients with a reduction or cessation of AP at D15 or D60; evaluation of QoL and AP characteristics at D0, D15, D30, D45, and D60 with MPN-SAF and AP questionnaires, respectively; modification of plasmatic concentrations of cytokines and neuropeptides at D0, D15, D30, and D60; and modification of epidermal innervation density and pruriceptor expression at D0 and D15.

**Discussion:**

The APHYPAP trial will examine the efficacy of aprepitant vs hydroxyzine (reference treatment for AP) to treat persistent AP in MPN patients. The primary objective is to demonstrate the superiority of aprepitant vs hydroxyzine to treat persistent AP of MPN patients. The treatment received will be considered efficient if the AP intensity will be reduced at 3/10 or below on VAS after 14 days of treatment. The results of this study may provide a new treatment option for this troublesome symptom and also give us more insights in the pathophysiology understanding of AP.

**Trial registration:**

APHYPAP. NCT03808805, first posted: January 18, 2019; last update posted: June 10, 2021. EudraCT 2018-090426-66

## Administrative information

Note: the numbers in curly brackets in this protocol refer to SPIRIT checklist item numbers. The order of the items has been modified to group similar items (see http://www.equator-network.org/reporting-guidelines/spirit-2727-statement-defining-standard-protocol-items-for-clinical-trials/).

**Table:** 

Title {1}	Rationale and design of the multicentric, double-blind, double-placebo, randomized trial APrepitant versus HYdroxyzine in association with cytoreductive treatments for patients with myeloproliferative neoplasia suffering from Persistent Aquagenic Pruritus. Trial acronym: APHYPAP
Trial registration {2a and 2b}.	ClinicalTrials.gov: NCT03808805 First posted: January 18, 2019 Last update posted: June 10, 2021 N°EudraCT: 2018-090426-66
Protocol version {3}	Study protocol version 5.0, 08/20/2021
Funding {4}	PHRCi Grand Ouest (Programme Hospitalier de Recherche Clinique inter-régional- Grand ouest) (2017).
Author details {5a}	Le Gall-Ianotto C^1,2^, Verdet R^3^, Nowak E^3^, Le Roux L^4^, Gasse A^4^, Fiedler A^**5,**6^, Carlhant- Kowalski D^7^, Marcorelles P^2,8,9^, Misery L^1,2^ and Ianotto JC ^10,11^ ^1^ (corresponding author) Department of Dermatology, University Hospital of Brest, France; ^2^ Univ Brest, LIEN, Brest, France; Address: 22 avenue Camille Desmoulins, 29200 Brest. Email: christelle.ianotto@chu-brest.fr; ^**3**^ Research and Innovation Department, University Hospital of Brest, Brest, France; ^4^ Clinical investigation center, CIC, Inserm 1412, University Hospital of Brest Brest, 29200, France; ^5^ Department of Pharmacy, University Hospital of Brest, France;^6^ PPRIGO (Production Pharmaceutique pour la Recherche Institutionnelle du Grand Ouest), Brest coordinator, France; ^7^ Pharmacovigilance Unit of Research and Innovation Department, University Hospital of Brest, France; ^8^ Brest Biological Resources Center BB-0033-00037, University Hospital of Brest, France; ^9,^ Department of Pathology, University Hospital of Brest, France; ^10^ Department of Clinical Hematology, University Hospital of Brest, France; ^11^ France Intergroup of Myeloproliferative neoplasms (FIM)
Name and contact information for the trial sponsor {5b}	CHRU de Brest – Direction de la Recherche et de l’Innovation – 2 avenue Foch – 29609 Brest cedex- France.
Role of sponsor {5c}	The funding instance has no role in the study design, data collection, data analysis and interpretation, writing the report or decision to submit the report for publication. Sponsor has a role of monitoring of the trial

## Introduction

### Background and rationale {6a}

BCR-ABL1-negative myeloproliferative neoplasms (MPNs) are chronic hematological diseases resulting from a clonal abnormality of the hematopoietic stem cells. They include mostly three diseases: essential thrombocythemia (ET), myelofibrosis (MF), and PV. They are secondary to the acquisition of genetic mutations, the main of which is *JAK2*V617F (> 95% in PV and between 50 and 60% in ET and PMF), inducing a medullary and then blood cellular hyperplasia that is responsible for the main risks associated to MPNs (arterial/venous thrombosis and hemorrhages). More recently, other mutations have been described in ET and PMF (CARL, MPL…) [1].

Among the physical symptoms dominating the management of MPN patients, the aquagenic pruritus (AP) is the most troublesome symptom described by the patient. AP is a diffuse itching sensation that develops after water contact at any temperature. It usually occurs immediately (1 to 5 min) after water contact and can last up to 10–120 min without any visible skin changes. AP is classically associated with PV where at least 30% of PV patients complain of it, but we have recently described that it is not only associated with PV but also can be present in 10% of ET and PMF patients with different clinical characteristics [2]. Furthermore, we have demonstrated that ET patients with AP were more symptomatic and had more proliferative features than the others; furthermore, during the follow-up, the patients experienced more thrombotic events and a 3-fold increase of phenotypic evolutions [3]. We also showed that the presence of AP was not correlated to the JAK2 mutations [3].

AP could often induce psychological disorders that could lead to a reduction of their participation in physical and social activities and consequently aggravates significantly alterations of quality of life (QoL) [4–7]. The hemogram normalization by the classic cytoreductive treatments (hydroxyurea, pipobroman, pegylated α2a interferon, anagrelide) induces little clinical response on AP that is then defined as refractory [2].

Since several years, a targeted treatment, the ruxolitinib or Jakavi®, a non-specific Jak2 inhibitor, received approval in the MFP treatment, due to a very strong efficacy in the reduction of the splenomegaly as well as constitutional symptoms as pruritus [8–10]. Its main mechanism of action would be the regulation of plasmatic levels of some cytokines [11, 12]. Recently, it received the approval in the second line in the treatment of PV and could be a precious actor in the treatment of AP. However, its expensive price (> 3000 euros/month/patient) and the lack of recommendation about its use in therapeutic association to only treat AP are clearly a brake to this one indication.

Due to the ignorance of the pathophysiology and the lack of pathophysiological studies, there is no efficient symptomatic treatment of AP [13]. Some arguments based on the direct study of skin (innervation, cutaneous barrier) and on communication between skin and blood could however be advanced.

#### Cutaneous nerve fibers (CNF) and pruritogens

Pruritus is transmitted by free epidermal nerve endings [14, 15]. These CNF contain neuropeptides such as substance P (SP), calcitonin gene-related peptide (CGRP), vasoactive intestinal peptide (VIP), and gastrin-releasing peptide (GRP), known to be inducers and potentializers of pruritus. For the study of the role of nerve fibers in the pathophysiology of AP, two parameters have to be considered: their epidermal density and the paracrine expression levels of neuropeptides/neuropeptide receptor systems. Recently, the sensitive skin, a syndrome associated with pruritus, has been characterized as a small fiber neuropathy [16–18] while, in atopic dermatitis (AD) and psoriasis, two pruritic dermatoses, an increase of GRP+, SP+, VIP+, and CGRP+ cutaneous fiber nerves has been clearly demonstrated [19–21]. These data indicate that pruritus could be associated with either a down or an up-regulation of cutaneous innervation. In 2003, a study performed on a woman with aquadynia has shown an increase of VIP-positive cells in the epidermis [22]. This neuropeptide has also been involved in AD, with the observation of an increase of cutaneous level [23]. In psoriasis, the increase of SP levels has been correlated to an overexpression of the SP/NK-1R system within the psoriatic area [24].

The assessment of epidermal nerve fiber density and expression of associated neuropeptides/neuropeptide receptors in AP has never been realized. The efficacy of some treatments such as phototherapy (UVA or UVB) or the topical application of capsaicin cream was described in some AP cases which could be explained by the destruction of these epidermal fibers [25–29].

Numerous pruritogens are released in the epidermis or sub-epidermis areas as prostaglandins, serine-proteases, cytokines...by different cellular types recruited in MPN patients [30]. So, these pruritogens are in close contact with the free nerve endings and could activate them and induce the release of their contents as SP and CGRP that act as a key inducer and potentializer of pruritus. The immunohistochemistry analyses would permit to better understand the involvement of the innervations in this pruritus induction.

#### Circulating cytokines

MPNs could be considered as inflammatory diseases with important quantities of pro-inflammatory cytokines and chemokines released in the blood flow [31–34]. Hematopoietic cells from MPNs show a sensitivity and an exacerbated response to cytokines and growth factors (EPO, GM-CSF, IL-3, IGF1…). This paracrine and/or autocrine stimulation induces an amplification of cellular proliferation, an increase of clinical symptoms, and an increase of the thrombotic risk [32–37]. Recently, two studies from Da Costa Cacemiro et al. shown (1) a higher rate of some cytokines and chemokines in MPN patients (PV, ET, and MF) as in healthy subjects, (2) a different cytokinic profile between the different MPNs that permits to help the differential diagnosis between PV and secondary polycythemia, and (3) that the mutational status (JAK2V617F + or −) had also an impact on the expression profile of cytokines [31]. Among these profiles described in the different MPNs, IL-1β, IL-4, IL-6, IL-10, IL-17A, and IFN-γ released by Th17 have been found [32, 38].

Cytokines among which the Th2 cytokines are known to be involved in the pathophysiology of pruritus associated or not to a dermatologic disease. Hence, the cytokines as IL-4, IL-5, IL-10, and especially IL-31 (a cytokine strongly involved in pruritus of AD) have been described in the pathophysiology of pruritus in cutaneous T-cell lymphoma [39–41]. But, too few data exist about the involvement of these cytokines and/or another one in the pathophysiology of AP. Il-31 is particularly interesting. Involved in the neurite outgrowth, it is also described in the pathophysiology of pruritus in atopic dermatitis and in Hodgkin lymphoma and cutaneous T lymphoma [42–44]. Thus, Ishii et al. in 2009 have shown that patients with PV and suffering from an AP had a plasmatic level of IL-31 more elevated than in patients without AP [39]. In 2010, the Gangemi team showed that there was no correlation between the plasmatic levels of IL-22, IL-23, and IL-10 and the presence of AP in PV patients, but the number of patients was very only ten [45].

Due to the involvement of these cytokines/chemokines in the pathophysiology of MPNs in one hand and in the induction of pruritus in different pathologies in the other hand, the systematic study of the cytokinic expression profile in MPN patients with AP appears to be important.

#### Therapeutics

Due to the absence of a real understanding of the pathophysiology mechanism of AP, numerous medications have been described in the literature with very fluctuating success depending on patient and treatments [11, 46, 47]. In 2012, a group of dermatologists having an expertise in chronic pruritus has recommended the use of hydroxyzine, an anxiolytic with antihistaminic and anticholinergic properties, for the treatment of AP in PV as an interesting alternative to relieve this symptom without resolving it. However, a placebo effect has been evoked by this group [13, 48].

As we said previously, the ruxolitinib (JAKAVI®) used in MF provides a good response in the relief of pruritus for a majority of patients. Recently, it received the approval to treat PV patients resistant or intolerant to hydroxyurea (HU) and showed also good action on AP [7, 9]. But, for PV patients having a good tolerance to HU and for who the cytoreductive medication is efficient to control the hemopathy, the use of ruxolitinib is not a good therapeutic strategy. Furthermore, this drug has not received the authorization for ET treatment. So, we think that it is important to bring another therapeutic solution to these patients. That is what we will propose in this study by using aprepitant, to find a therapeutic alternative less contraignive than a complete modification of the medication and less expensive than the use of JAKAVI®.

Aprepitant, firstly developed as an antidepressive, is actually used in cancerology as an efficient antiemetic. It is a specific antagonist of the NK1R, a receptor for SP and neurokinins A and B. So, aprepitant has been successfully tested in chronic pruritus associated with dermatological, hematological, or systemic diseases as well as drug-induced pruritus. Thus, its efficacy has been demonstrated in the treatment of pruritus in the case of nodularis prurigo [49]. In hemopathies, reduction of pruritus intensity to 80% (self-evaluation of intensity on a visual analog scale (VAS)) has been described in cutaneous T lymphoma or in Hodgkin lymphoma [50–54]. Furthermore, its efficacy has been shown to treat a refractory pruritus in the case of lymphoproliferative syndrome [55]. These results are not very surprising due to the fact that aprepitant targets the way of SP. However, its effect has never been evaluated in the treatment of AP in MPNs.

### Rationale

In this context, the AP is a clinical troublesome symptom with numerous physical and psychological complications and a real impact on social life. Despite a partial or complete hematological response due to their hematological treatment, more than 50% of patients describe a persistent AP 5 years after the MPN diagnosis. At the time of writing the protocol, there are no specific biological and/or clinical studies on AP in MPN patients or effective treatment to treat AP. Actually, the ruxolitinib appears to be an effective treatment to ameliorate the physical symptoms present in MPN but the price is an important brake as well as the fact that it is not available for all MPN as the first line of treatment. So, for all these reasons, aprepitant appears to be a good therapeutic alternative for the treatment of AP in MPN which has never been studied so far in this context.

### Objectives {7}

The objectives of this study combine both a clinical goal with the evaluation of the efficacy of aprepitant versus hydroxyzine in the treatment of a persistent AP for patients suffering from MPN (PV or ET or MF) and a biological aim to elucidate the pathophysiology of this symptom.

So, the primary objective is to demonstrate the superiority of aprepitant compared to hydroxyzine to treat persistent AP of MNP patients. The received treatment will be considered as effective with a reduction of AP intensity below (or equal) at 3/10 on the VAS after 14 days of treatment.

The secondary objectives will evaluate:

- The number of patients with a total relief of AP
- The time to response
- The duration of response during and after the end of the treatment
- The impact of the treatments on other general symptoms (asthenia, fever, nocturnal sweat)
- The quality of life before, during, and after the treatments
- The tolerance of drugs by identification of side effects
- The impact on the hematological response (European LeukemiaNet criteria)
- The study of the circulating cytokines before, during, and after the treatment
- The study of cutaneous characteristics of patients suffering from AP by skin biopsies before and after treatment

### Trial design {8}

The APHYPAP trial is an exploratory, multicentric, randomized, double-blind, double-placebo, phase III study comparing the efficacy of once daily aprepitant versus hydroxyzine to treat a persistent AP in patients with MPNs (ET, PV, or MF) despite their hematological therapy. Subjects will be stratified by investigational site and randomization will be performed with a 1:1 allocation.

## Methods: participants, interventions, and outcomes

### Study setting {9}

**Fig. 1:**
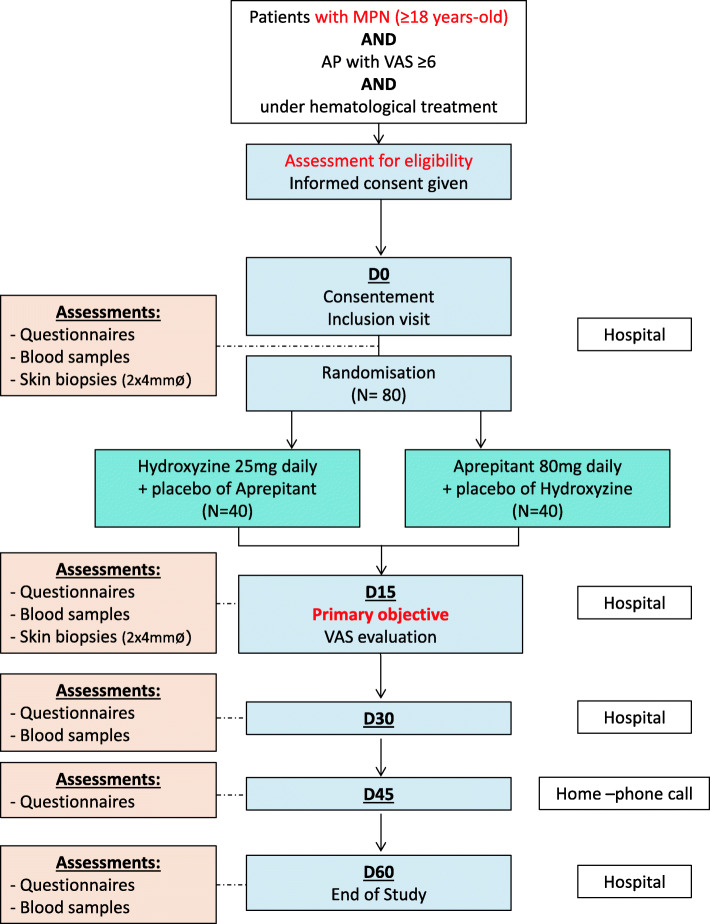
Flow chart of the study

The protocol will be conducted on 8 sites located in France (Angers, Brest, Caen, Grenoble, Lyon, Nantes, Rennes, and Quimper) under the direction of the university hospital of Brest. All MPN patients aged 18 years and older suffering from an AP refractory to hematological therapy since at least 6 months will be considered for participation (Fig. 1).

### Eligibility criteria {10}

#### Inclusion criteria

- Adult patients (men or women) suffering from one type of MPN (ET, PV, or MF),

AND - Treated by hydroxyurea, pipobroman, anagrelide, pegylated-IFN α2a, ruxolitinib, or phlebotomies for more than 6 months

AND - Suffering from an aquagenic pruritus despite their cytoredutive treatment AND - With a pruritus intensity higher or equal to 6 on the VAS (score from 0 to 10)

- For women of childbearing age, a negative pregnancy test (hormonal or commercial test)
- Patients who gave their written consent for participation in the study.

#### Exclusion criteria

- Patients with a physical or psychological disability to sign the informed consent
- Patients only treated by aspirin for their MPN
- Patients already included in another therapeutic study
- Patients suffering from a dermatological disease in which a diffuse pruritus may be present (psoriasis, atopic dermatitis, nodularis prurigo…)
- Patients already on antidepressant and/or anti-anxiety treatment (criterion leaves to physician’s discretion)
- Patients having a contraindication or hypersensibility to aprepitant or hydroxyzine or to one of their excipient
- Lactose intolerance
- Pregnant or lactating women

### Who will take informed consent? {26a}

Potential patients for the study receive oral information during a routine consultation with their hematologist trained in Good Clinical Practice (GCP). The written information containing all the information concerning the protocol will be given at the same time. After time for consideration, written informed consent will be collected by physicians and research team for patients who want to participate.

### Additional consent provisions for collection and use of participant data and biological specimens {26b}

All information concerning the biological specimens taken, storage, and use during this study will be notified in the informed consent. The signature of this form signifies that the patient is aware of the future of its biological samples and agrees with the procedure. But, at any time of the protocol and even after the end of the study, the patient could reverse his decision and all the biological samples will be destroyed.

### Interventions

#### Explanation for the choice of comparators {6b}

The experimental molecule tested here is aprepitant as an effective antipruritic drug.

The comparator chosen in this study is hydroxyzine which is considered as the best treatment option to treat AP by dermatologists despite low-level evidence [13].

#### Intervention description {11a}

##### Routine care

All participants are MPN patients who are treated for their hematologic disease by either hydroxyurea, pipobroman, anagrelide, pegylated-interferon α2a, ruxolitinib, or phlebotomies for at least 6 months; no change in the type of treatment or dose of the treatment will be made during their participation in this protocol.

##### Study treatment

The intervention will be the oral administration of a daily dose of 80 mg of aprepitant plus one tablet of a placebo to match to hydroxyzine, starting on day 1 until day 14. The comparator will be the oral administration of a daily dose of 25 mg of hydroxyzine plus one capsule of placebo to match to aprepitant, starting on day 1 until day 14. Because of the drowsiness of hydroxyzine, it will be recommended to take the treatments whatever the arm of treatment, in the evening.

##### Aprepitant

Aprepitant (EMEND®, Merck Sharp & DohmeDohme B.V., Waarderweg 39, 2031 BN Haarlem, Pays-Bas) is an antagonist of the specific receptor of substance P, the neurokinin-1 receptor. It is classically registered as an antiemetic agent for chemotherapies. The capsule of aprepitant 80 mg being marked, they will be hidden in larger capsules with no mark.

The placebo of aprepitant matches to capsules of neutral homeopathic granules (constituted by saccharose 85% and lactose 15%) which will be hidden in the same larger capsules as aprepitant.

##### Hydroxyzine

Hydroxyzine (Atarax®, UCB PHARMA SA, Colombes, France) is conditioned in size 4 red and white capsule dosed to 25 mg.

The placebo of hydroxyzine matches to size 4 red and white capsules with monohydrate lactose and cochineal camin.

All of the intervention treatments and their placebos are made indistinguishable to respect the double-blind.

The exact number of capsules to follow the 14 days of treatment will be given to each patient. Drugs must be stocked in a dry place with a temperature not exceeding 25 °C and out of care of children.

### Criteria for discontinuing or modifying allocated interventions {11b}

Patients may be withdrawn from the study or their treatment discontinued at any time of the protocol and for any of the following reasons:

- Withdrawal of informed consent
- Patient refusal or non-compliance to the protocol
- Use of concomitant medication which according to the judgment of the investigator may interfere with the objective of the study
- Occurrence of an unexpected serious adverse effect and in particular in case of aggravation of AP
- At the specific request of the sponsor

#### Replacement of individual subjects after withdrawal

Owing to the characteristics of the treatment and to the short length of the study (2 months), a premature withdrawal of the protocol is not expected apart from the stop of the treatment because of aggravation of AP. Any aggravation of AP will be considered as a failure of the treatment and will be noted in the analysis of the primary endpoint of the study.

If the patient withdraws his consent before the beginning of the treatment (before day 0), data will not be analyzed. On the other hand, if patient withdrawal is known after day 1, all the available data will be analyzed.

### Strategies to improve adherence to interventions {11c}

At the beginning of the protocol, D0, each included and randomized patient will receive one box containing 14 capsules of aprepitant 80 mg/placebo and one box of 14 capsules of hydroxyzine 25 mg/placebo. At the end of the treatment plus 1 day, D15, the remaining capsules will be counted by the investigator or by the research technician of each center. It signifies that all the blisters even if empty must be kept in the box. All the treatment boxes must be returned to the pharmacy of the investigator center at the end of each treatment. These boxes will be conserved until the promoter establishes the certificates of destruction.

### Relevant concomitant care permitted or prohibited during the trial {11d}

#### Medication or therapies permitted during the study

Subjects are allowed to use all the drugs which are prescribed by their hematologist: hydroxyurea (Hydrea®), pegylated-IFN α2a (Pegasys®), anagrelide (Xagrid®), pipobroman (Vercyte®), ruxolitinib (Jakavi®), aspirin, and anticoagulant treatments (anti-vitamin K, new anticoagulant, Clopidogrel).

A special attention must be given to antidepressive, atropininic, and other molecules with some atropinical side effects. Their continuation during the protocol will be decided by the hematologist.

#### Medication or therapies prohibited during the study

Due to their interaction with either hydroxyzine or aprepitant, the following drugs are prohibited during the treatment phase of the study.

##### For aprepitant

- Pimozide, terfenadine, astemizole, cisapride
- Molecules activating the CYP3A4: rifampicine, phenytoine, carbamazepine, phenobarbital
- Molecules inhibiting the CYP3A4: ritonavir, ketoconazole, clarithromycine, telithromycine
- Plant preparations with St. John’s wort (*Hypericum perforatum*)

##### For hydroxyzine

All drugs containing alcohol (Cefamandole, cefoperazone, chloramphenicol, glibenclamide, glipizide, tolbutamide, disulfirame, furazolidone, griséofulvine, nitro-5-imidazole, procarbazine).

### Provisions for post-trial care {30}

Standard care is provided after participants have finished the study treatment phase.

An insurance has been subscribed for the participants in this clinical trial.

### Outcomes {12}

#### Primary endpoint

The primary study endpoint will be the reduction of pruritus intensity below (or equal to) 3/10 evaluated at day 15 by VAS.

#### Secondary endpoints

The secondary endpoints will be:

- Number of patients with a pruritus intensity below (or equal) at 3/10 on the VAS at D60
- Number of patients with cessation of pruritus (intensity at 0/10 on the VAS) at D15
- Number of patients with cessation of pruritus (intensity at 0/10 on the VAS) at D60
- Number of days to obtain an intensity of pruritus at 3/10 on the VAS from D1 to D60
- Number of days the visual analog scale (VAS) is below (or equal) at 3/10
- Type of adverse event occurring during the therapeutic association during the 15 days of treatment
- Total number of prematurely discontinued treatments for all subjects at D15
- Number of patients with hematologic remission from D1 to D60 evaluated by complete blood count: hematocrit < 45%, leukocytes < 10 giga/l, and platelets < 400 giga/l
- Evaluation of quality of life at D0 (day of inclusion), D15 (after treatment), D30, D45, and D60 by completion of the MPN-SAF questionnaire (Myeloproliferative Neoplasm Symptom Assessment Form calculated as the mean score for 10 items. Questions focus on fatigue, concentration, early satiety, inactivity, night sweats, itching, bone pain, abdominal discomfort, weight loss, and fevers) [56]
- Evaluation of pruritus at D0 (day of inclusion), D15 (after treatment), D30, D45, and D60 by completion of the PASYMPLE (evaluation of pruritus with 7 questions about occurrence, timing, intensity, and location of pruritus) questionnaire [2]
- Quantification of plasmatic concentration change of cytokines and neuropeptides analyzed at D0, D15, D30, and D60
- Study of cutaneous characteristics by skin biopsies (epidermal innervation density, pruriceptor expression) by immunohistochemistry at D0 (day of inclusion) and D15 (after treatment)

### Participant timeline {13}

See Fig. 2.

**Fig. 2:**
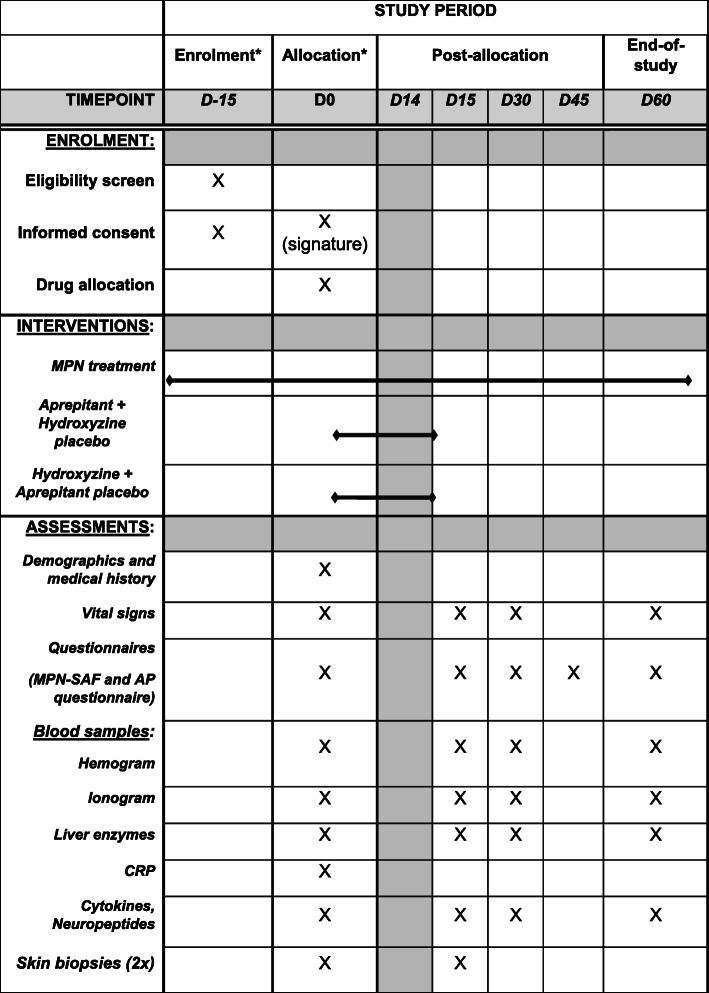
Overview of study and assessments. *Consultations that could be merged

### Sample size {14}

Our assumption is that the expected success rate (VAS ≤ 3) at D15 in the control group (hydroxyzine) will be at most 30% versus 70% at least in the experimental group (aprepitant). Thirty-six patients are required in each arm to validate this hypothesis (Casagrande and Pike exact formula), with a two-sided test, an *α* of 5%, and a power of 90%.

To anticipate potential missing data, a total number of 40 subjects per study arm will be included so a total of 80 subjects is expected in this study.

Eight French departments of Hematology will be opened for this trial.

### Recruitment {15}

The study team in each center will be responsible for identifying potential participants. Patients will be orally informed of the protocol during a routine consultation completed by the delivery of the written information. If eligible and agreed to participate, patients will have a new date of consultation; the written informed consent signed by the participant will be collected and the patient may be included in the protocol.

## Assignment of interventions: allocation

### Sequence generation {16a}

Participants will be randomly allocated to either the experimental arm (aprepitant/placebo of hydroxyzine) or the control arm (hydroxyzine/placebo of aprepitant) with a ratio 1:1 using computer randomization. The randomization will be stratified by site.

The randomization procedure with treatment allocation will be done by the study team member using the “Capture System,” a Web-based system that will be used for data entry.

### Concealment mechanism {16b}

The Web-based used for randomization will ensure the allocation concealment.

### Implementation {16c}

The allocation sequence generation will be embedded in Capture System software. The investigator doctor of each center will enroll patients and randomize them by using the same software.

## Assignment of interventions: blinding

### Who will be blinded {17a}

The double-blind randomized experimental scheme of the study allows to limit the statistical bias as well as the intervention bias. So, active treatments (aprepitant or hydroxyzine) and their placebos will be allocated in blind: neither the patient nor the investigator doctors, the nurses, or the clinical research associate (CRA) will know the allocated treatment.

Furthermore, the presentation and the packaging of active treatments and placebos will be identical.

### Procedure for unblinding if needed {17b}

The unblinding will be carried out systematically at the end of the study.

Information on the correspondence between the treatment arm and the treatment number will be held by the central pharmacy of the CHRU de Brest and the data management unit.

Study team members and healthcare providers do not have access to the treatment allocation code. However, if an investigator wants to introduce a medication that should be not taken at the same time to one of the study treatments, the blinding code will be broken.

No expected adverse event in the study will require an emergency unblinding. In case of suspected unexpected serious adverse reaction, the sponsor will declare the serious and unexpected adverse reaction to the health authorities and to the CPP after having unblinded the investigational medicinal product

## Data collection and management

### Plans for assessment and collection of outcomes {18a}

#### Vital signs and physical examination

Depending on the clinical practice of each hematologist physical examination and vital signs as systolic and diastolic blood pressure, heart pulsations, and tympanic temperature could be realized at the inclusion visit, D0, and at each visit during the study (D15, D30, and D60). However, these data will not be notified in the eCRF.

#### Blood samples

During the study, blood samples for clinical analyses (chemical and hematological) and translational studies (plasmatic cytokines/chemokines and neuropeptides) will be collected at baseline (D0) and during the study at D15, D30, and D60.

#### Questionnaires

MPN symptoms, intensity, and characteristics of the AP will be assessed at baseline (D0), after the experimental procedure (D15), and all along the study (D30, D45, and D60). Except for the D45 assessment that will be realized by a phone call, the questionnaires will be completed during the hospital visits. For the assessment of MPN symptoms, the concise, valid, and accurate MPN-SAF TSS questionnaire will be used [57]. For the pruritus questionnaire, the PASYMPLE questionnaire will be used [2].

Furthermore, all along the study, patients must fill a logbook (received at the inclusion visit) in which they must refer each water contact they have, induction of AP if it is the case, its intensity (VAS), and timing of the crisis. All the data are included in the eCRF.

#### Skin biopsies for translational study

**Fig. 3:**
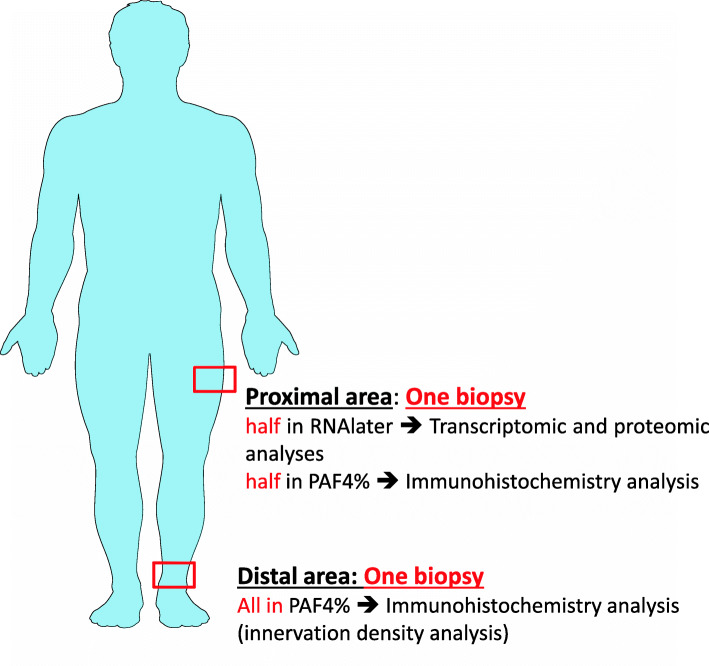
Methodology for skin biopsies

At D0 and D15, included patients will be addressed to the dermatological department of the hospital in which 2 skin biopsies of 4 mm ø will be realized under local anesthesia and will follow the procedure described in Fig. 3. The location of biopsies follows the protocol of small fiber neuropathy diagnosis: one at the distal location on the tight, one at the proximal location, and 20 cm above the malleolus. This procedure will allow to determinate intraepidermal nerve density fiber (IENF) [57]. The distal biopsy fixed in PAF4% will be used for the IENF analysis. The proximal biopsy will be cut in two: one part put and conserved in RNAlater® will be used for transcriptomic analysis of pruriceptors, and the second part for the immunohistochemistry expression analysis of markers known to be involved in pruritus. A home nurse will remove the stitches few days later.

#### Treatments

Patients will receive their 14-day treatment at the inclusion visit (D0) and start it the same day, in the evening.

### Plans to promote participant retention and complete follow-up {18b}

There are no specific measures to promote participant retention. The study is only 2 months long with a sequence of visit every 15 days. For all subjects who discontinued the protocol, assessments can be included in the analysis.

### Data management {19}

At the time of inclusion, each participant will receive an individual, site-specific study code. All study data will be collected in an electronic case report form using Web-based data capture software (eNNOV Clinical). Data will be notified in the eCRF as soon as they will be collected. Each missing data will be codified.

This eCRF will be accessible in each center thanks to a Web access. The central data manager checks data entry of all sites and ensures that inaccurate or missing data are addressed as soon as possible. All data will be immediately checked thanks to coherence controls. So, any modification of value will be validated. Risk-adjusted monitoring will be performed by a clinical research associate (independent staff of the research team that is mandated by the sponsor) and will consist of checking the informed consent form, completeness of eCRF, and source data verification for all patients.

### Confidentiality {27}

All people involved in this study and having access to the nature of experimental drugs, information relative to participants at the study, and research results will take all necessary caution to keep them strictly confidential. Furthermore, investigators and all people acting in this study are subject to professional secrecy. Collected data will be kept strictly confidential and stored in accordance to General Data Protection Regulation and Good Clinical Practice. To that end, collected data and sent to the promotor will be made anonymous. Only the first letter of the name and the first letter of the surname of the participant will be registered associated to a specific study code indicating the order of inclusion of the subject. Confidential data are accessible only to the data management department of the hospital of Brest.

### Plans for collection, laboratory evaluation, and storage of biological specimens for genetic or molecular analysis in this trial/future use {33}

Blood samples will be taken at baseline (D0) and during the study at D15, D30, and D60; one part will be used for clinical follow-up (blood chemistry); and plasma will be collected on the other samples for a translational study. The blood chemistry panel consists of sodium, potassium, calcium, uric acid, creatinine, phosphorus, albumin, total bilirubin, liver enzymes (alkaline phosphatase ALT, AST, LDH, gamma-GT, lipase, ferritin), and glucose. CRP quantification will only be realized at D0. The hematological panel consists of hemoglobin, hematocrit, white blood cell count and differential, and thrombocytes. For the translational study, aliquots of 250 μl of plasma will be realized, frozen, and stocked at −80 °C in the Clinical Investigation Center of the Hospital of Brest. For this study, plasmatic cytokines, chemokines, and neuropeptide concentrations will be assessed by Multiplex assay (Luminex technology) and ELISA tests, respectively.

The remaining plasma samples, stocked in the Clinical Investigation Center of the Hospital of Brest, can be used for other research while respecting the procedure for the provision of samples from a clinical trial, edited by this institution.

Concerning the skin biopsies realized at D0 and D15 for each patient (one in PAF 4%, one cut in two with one part in RNAlater®, and the second in PAF 4% as described in Fig. 3), they will be frozen and stocked at −80 °C in the Brest Biological Resources Center BB-0033-00037 (“CRB Santé du CHRU de Brest”). The distal biopsy fixed in PAF4% will be used for the IENF analysis. This will allow to determine if AP induction may be correlated to a default of innervation. The biopsies put in RNAlater® will be used for a transcriptomic analysis of pruriceptors, semaphorins, and proteins involved in keratinocyte/neuron communication. These analyses may be supplemented by the evaluation of the protein expression levels by immunohistochemistry. This will be realized on the second part of the biopsy fixed in PAF4%.

The remaining samples will be conserved at −80 °C in the CRB for further analyses if needed.

The aim of the translational study is to clarify the pathophysiology of AP.

## Statistical methods

### Statistical methods for primary and secondary outcomes {20a}

Primary efficacy analysis will be performed as intention-to-treat (ITT). The percentage of patients for which the VAS will be ≤3 (from 0 to 10) at D15 will be compared in the two groups with a chi test or a Fisher test if needed.

The following comparisons will be next performed according to the hierarchical analysis principle (points to consider on multiplicity issues in clinical trials, EMEA 2002, CPMP/EWP/908/99):

- Comparison of percentage of patients for which the AP intensity (VAS) is ≤3 points on 10 at D60 (chi-squared test)
- Comparison of the mean scores obtained at the MPN-SAF questionnaire at D60 with adjustments on baseline value (D0)

Exploratory, the AP intensity evolution during the follow-up will be modeled using a mixed model for repeated data and compared between the two groups, as the evolution of the score obtained with the MPN-SAF questionnaire.

### Interim analyses {21b}

No interim analysis has been planned for this study.

### Methods for additional analyses (e.g., subgroup analyses) {20b}

No subgroup or adjusted analysis has been planned in this study.

### Methods in analysis to handle protocol non-adherence and any statistical methods to handle missing data {20c}

Study outings due to inefficiency will be considered as a treatment failure. In all the cases mentioned, missing data will be described in terms of numbers and corresponding percentages.

### Plans to give access to the full protocol, participant-level data, and statistical code {31c}

For any supplementary documentation other than the information given in the manuscript, the corresponding author will answer to any request.

## Oversight and monitoring

### Composition of the coordinating center and Trial Steering Committee {5d}

The coordinating center team consists of the principal investigator, associated investigators, clinical research technicians, and research nurses.

The Trial Steering Committee (TSC) consists of the principal and coordinating investigator, the methodologist and statistician of the trial, two associated investigators, and an associate scientist that will oversee the project and meet regularly.

### Composition of the data monitoring committee, its role, and reporting structure {21a}

An independent data safety monitoring committee consists of 4 independent members specializing in hematology, dermatology, internal medicine, and hemovigilance, not involved in the study and meets first at 20 patients included in the protocol. Then, the frequency of subsequent meetings will be determined according to the events and the pace of inclusions. The committee analyzes the trial safety, the presence or not of adverse events, the good compliance of the protocol, and if it is safe to continue the study with or without protocol modifications or if there is any reason to stop the study.

### Adverse event reporting and harms {22}

Adverse events (AE) reported by the patient or by the investigator will be recorded and scored according to the NCI Common Terminology Criteria for Adverse Events (CTCAE). If AE happen during the experimental treatment period, all the details will be notified in the eCRF as time of occurrence, clinical symptoms and signs, degree, duration, and causal relationship with the treatment.

In case of serious adverse events (SAE), the investigator has to declare to the promoter within 24h after the first knowledge of the SAE. The investigator may stop immediately the treatment if it is considered in the best interest of the patient. The imputability of the SAE with the experimental has to be established. SAE with a doubtful, possible, probable, or highly probable with aprepitant or hydroxyzine will be considered as associated to them. If SAE are unexpected, they will be designed as suspected serious adverse reactions (SUSARs). In this case, the promoter has to declare them to the Eudravigilance, to Agence Nationale de Sécurité du Médicament (ANSM), and ethical committee and other investigators. This will occur not later than 15 days after the first knowledge of the AE. In case of fatal or self-threatening cases, the delay will be reduced to maximal 7 days and 8 days supplementary for the report completion.

Once a year, the promoter will send a report with the complete list of SAE that could be associated to the experimental medication including expected and unexpected effects, and a precise and critical analysis of the safety of participants included in the study.

### Frequency and plans for auditing trial conduct {23}

The steering committee of the study will systematically meet every 12 months after the beginning of the trial.

The independent monitoring committee will meet at 20 included patients and then when it is necessary and will analyze the progress of the study, the inclusions, and the occurrence and grade of each reported AE. They will transmit their recommendations and advices to the steering committee after each evaluation. The steering committee will then decide whether or not to continue the study.

Concerning the centers involved in the study, at the end of the inclusions, the clinical research associate (CRA) will audit on-site the good process of the protocol.

### Plans for communicating important protocol amendments to relevant parties (e.g., trial participants, ethical committees) {25}

All the modifications needing substantial amendments will be discussed within the TSC and submitted to trial control instance (ANSM, ethical committee). The modifications must be accepted by these instances. Once accepted, modifications will be notified in trial registries and documents. Patients included in the study will be informed on important protocol modifications if personally relevant for them.

## Dissemination plans {31a}

The clinical and biological results of the study will be disseminated to all stakeholders, including clinicians, scientists, and patients. This communication on the research results will be done through publications in peer-review journals, oral or poster communication in (inter)national congresses, and symposia concerning both hematology and dermatology areas. Furthermore, communications will also be disseminated in (inter)national expert networks: in hematology with the FIM group (France Intergroupe des syndromes Myéloprolifératifs) and in dermatology with the IFSI group (International Forum for the Study of Itch). Patients will be informed of the end of the study and of results via a short letter that will be addressed directly to patients or via association for patients suffering from MNPs.

## Discussion

AP is described by MPN patients as the most troublesome aspect of their disease which has a real impact on their social life and QoL [5]. But, beyond that, its presence could have an impact on the occurrence of complications such as thromboses. Thus, while Gangat demonstrated that the presence of AP in PV patients was associated to a lower risk of arterial thromboses [58], we demonstrated in ET patients that the presence of AP was correlated with higher risks of thromboses and phenotypic evolution [3]. Thus, AP is not only an annealing symptom, but must be questioned at the time of diagnosis and considered in the management of the hemopathy. For most of the patients, the cytoreductive treatments allow a relief even a complete resolution of AP. Unfortunately, AP remains resistant for 33% of PV, % of ET, and % of PMF patients [2]. Considering that the pathophysiology of AP remains obscure, the patients begin then a therapeutic wandering to treat the symptom.

In 2012, the group of experts on chronic itch defined the use of hydroxyzine as the first choice to relieve AP in PV patients [13]. However, despite the fact that high levels of histamine due to the augmented number of basophiles have been suggested to trigger itch in PV [39], the limited evidence of antipruritic effects of antihistamines to treat AP suggested that antipruritic action of such molecules might be associated to a placebo effect. More specific and more efficient treatments are needed to treat AP. Since several years, the success of aprepitant to treat chronic itch in Hodgkin lymphoma, cutaneous T-cell lymphoma, and Sezary lymphoma described in studies may consider it a potential and efficient candidate to treat AP of MPN [50–55]. No existing clinical trial has analyzed its effects on AP. So, we propose to evaluate its efficacy to treat AP in MPN patients in a phase III, double-blind, double-placebo, randomized, multicentric, therapeutic clinical trial. The primary objective is to demonstrate the superiority of aprepitant vs hydroxyzine to treat persistent AP of MPN patients. The treatment received will be considered efficient if the AP intensity will be reduced by ≥3 points (visual analog scale (VAS)) after 14 days of treatment. The double-placebo is justified by the fact that the placebo effect is often described when treating chronic pruritus [13]. Very few side effects are awaited with either of the drugs; constipation is described for aprepitant and drowsiness for hydroxyzine that is why it will be recommended to take the drugs in the evening. Concerning the choice of the dose and the time of treatment, no clear experimental procedure was described in the literature for this purpose. The classic use of aprepitant in the antiemetic context described cycles of 3 days every 2 weeks of 125 mg D1/80mg D2/80mg D3 [59]. For pruritus treatment, different protocols have been reported. So, our posology was defined by confronting several publications treating chronic pruritus with aprepitant with success and discussion with the dermatologist [50, 60, 61].

In conclusion, with this study, we hope to show that aprepitant might be a simple and efficient treatment to relieve AP for MPN patients for which this symptom is the most troublesome aspect of their hemopathy. Furthermore, this study will give more insight into the pathophysiology of this very special pruritus with the evaluation of plasmatic cytokines and neuropeptides, the description of the epidermis innervation, and the analysis of the expression of many skin pruriceptors before and after treatment. If aprepitant proves to be effective in the relief of AP, it will be an important step in the management of MPN patients.

## Trial status

Protocol version 5.0, 08/20/2021

Status: recruiting (start: 16/04/2019), 8 centers involved are recruiting.

Approximate date when recruitment will be completed: 16/10/2022

## Abbreviations

AE: Adverse event
ANSM: Agence Nationale de Sécurité du Médicament
AP: Aquagenic pruritus
CPMP: Committee for Proprietary Medicinal Products
CTCAE: Common Terminology Criteria Adverse Effects
D: Day
eCRF: Electronic case report form
CRP: C-reactive protein
EMEA: The European Agency for the Evaluation of Medicinal Products
ET: Essential thrombocythemia
IFSI: International Forum for the Study of Itch
ITT: Intention-to-treat
FIM: France Intergroupe des syndromes Myéloprolifératifs
MNPs: Myeloproliferative neoplasms
MPN-SAF: MyeloProliferative Neoplasm Symptom Assessment Form
NK-1R: Neurokinin receptor 1
PV: Polycythemia vera
PMF: Primary myelofibrosis
QoL: Quality of life
SAE: Serious adverse event
SP: Substance P
TSC: Trial Steering Committee
VAS: Visual analogic scale
